# Poor glycemic control might compromise the efficacy of chemotherapy in non‐small cell lung cancer patients with diabetes mellitus

**DOI:** 10.1002/cam4.2750

**Published:** 2019-12-12

**Authors:** Xianghua Zeng, Cheng Xu, Jianan Cheng, Chengdu Sun, Zhongyu Wang, Zhihua Gong, Haixia Long, Bo Zhu

**Affiliations:** ^1^ Institute of Cancer Xinqiao Hospital of the Third Military Medical University Chongqing China; ^2^ Department of Oncology General Hospital of the Central Theater Command of the People's Liberation Army Wuhan Hubei Province China; ^3^ Chongqing Key Laboratory of Immunotherapy Xinqiao Hospital of The Third Military Medical University Chongqing China

**Keywords:** glycemic control level, non‐small cell lung cancer, platinum‐based chemotherapy, progression‐free survival, type 2 diabetes mellitus

## Abstract

**Background:**

Previous studies indicated that type 2 diabetes mellitus (T2DM) is related to an increased lung cancer risk, but its role in the prognosis of NSCLC remains conflicting. This study investigated the impact of blood glucose control on the outcomes in NSCLC patients with T2DM treated with platinum‐based doublets.

**Methods:**

Clinicopathological and survival data from 191 T2DM patients with advanced NSCLC, who received platinum‐based chemotherapy, were retrospectively analyzed. Based on the blood glucose conditions during chemotherapy, patients were classified into poor (n = 84) and good control (n = 107) groups. Progression‐free survival (PFS) was assessed using the Kaplan‐Meier method.

**Results:**

The median PFS among patients with good glycemic control [197.0 (95% CI: 136.3‐257.7) days] was longer than that among those with poor control [132.0 (95% CI: 112.5‐151.5) days] (*P* = .0003). Further subgroup analysis of lung squamous carcinoma and adenocarcinoma patients showed that the median PFS of the good control group was also significantly longer than that of the poor control group [179.0 (95% CI: 78.4‐279.6) days vs 125.0 (95% CI: 110.9‐139.1) days, *P* = .0014; 197.0 (95% CI: 124.3‐269.7) days vs 154.0 (95% CI: 129.9‐178.1) days, *P* = .0359; respectively]. The incidence rates of side effects were similar among patients with good glycemic control and those with poor glycemic control (all *P* > .05).

**Conclusions:**

Satisfactory glycemic control during platinum‐based chemotherapy might provide a survival benefit to T2DM patients with NSCLC. Further studies are warranted to confirm our findings.

## Graphical abstract

In non‐small cell lung cancer patients with type 2 diabetes mellitus, poor glycemic control may lower the efficacy of platinum‐based chemotherapy due to the shorter progression‐free survival, and so it is much important to strengthen glycemic control in these patients.

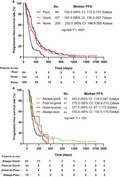

## 1 INTRODUCTION

Lung cancer has become one of the most common malignant tumors to threaten human health due to its high morbidity and mortality.^1^, ^2^ Non‐small cell lung cancer (NSCLC) accounts for more than 85% of all lung cancer cases.^3^ As the latest data showed, approximately 30% of NSCLC patients diagnosed at an advanced stage often miss the opportunity for radical surgery, with a median progression‐free survival (PFS) of about 8 months and a five‐year survival rate of only 15%.^4^, ^5^ For advanced‐stage patients without target gene mutations, platinum‐based and two‐drug chemotherapy regimens have long been the standard first‐line treatment, which might lead to a median overall survival of less than 10 months.^6^

Although the late diagnosis of NSCLC is widely recognized to be associated with worse survival, comorbidities, particularly type 2 diabetes mellitus (T2DM), are another major risk factor affecting the prognosis of patients with NSCLC in clinical practice.^7^, ^8^, ^9^ The results of a multivariate analysis showed that, patients with T2DM present a significantly increased risk of various types of tumors including lung cancer.^10^, ^11^, ^12^ The coexistence of T2DM in NSCLC patients has become more common, with an estimated proportion of 8%‐18%.^13^ Hyperinsulinemia, hyperglycemia and chronic inflammation in patients with T2DM may influence the progression and outcome of lung cancer.^14^, ^15^ However, a large number of studies have been concerned with the prognostic significance of preexisting diabetes mellitus (DM) or the fasting blood glucose (FBG) level in patients with NSCLC, not the glycemic control conditions.^10^, ^15^, ^16^ In addition, the influence of DM on the survival of patients with lung cancer remains controversial. Some studies have reported that preexisting DM was considered an independent inferior prognostic factor for overall survival and PFS, while others found that DM did not impact or even prolong lung cancer patients’ survival.^9^, ^17^, ^18^, ^19^ Accordingly, we deemed that glycemic control levels, not DM, might be an appropriate prognosis predictor during chemotherapy.

Considering all the findings above, we conducted this retrospective study to investigate the impact of the blood glucose conditions of T2DM on PFS in patients with unresectable and advanced NSCLC treated with platinum‐based doublet chemotherapy.

## 2 METHODS

### 2.1 Subjects

Eligible study subjects were recruited among patients admitted to Xinqiao Hospital, Chongqing, China, from January 2010 to May 2019, and analyzed retrospectively. All NSCLC patients with T2DM had histologically proven advanced‐stage (III or IV), and unresectable disease based on the International Association for the Study of Lung Cancer Staging Manual in Thoracic Oncology, version 7.^20^ All patients enrolled had received two or more cycles (defined according to local practice) of platinum‐based chemotherapy [containing gemcitabine, vinorelbine, a taxane (paclitaxel or docetaxel), or pemetrexed] after the diagnosis of cancer. Two hundred among the set of more than 2000 patients with advanced NSCLC, who received platinum‐based chemotherapy (and met the inclusion criteria set out in the study) but without T2DM, were randomly selected to comprise the control group.^21^

Specifically, patients enrolled were required to meet the inclusion criteria as follows: (a) a histological diagnosis of locally advanced and/or metastatic NSCLC (stage III or IV); (b) received no surgery, chemotherapy or radiotherapy before inclusion, and more than two cycles (defined according to local practice) of platinum‐based chemotherapy containing doublets after enrollment; (c) tumor growth should be assessed by chest computed tomography (CT), magnetic resonance imaging or ultrasound, and evaluated based on Response Evaluation Criteria in Solid Tumors (RECIST)^22^; and (d) the FBG levels before at least two cycles of chemotherapy were monitored. Patients were excluded if they: (a) concurrently had other cancers; (b) received surgery, targeted therapy or immunotherapy during chemotherapy; or (c) did not have evaluable radiological images.

The following baseline data were collected for analysis: age, sex, body mass index (BMI), tumor stage (TNM), histological classification, drinking status, smoking history, Eastern Cooperative Oncology Group performance status (ECOG PS), T2DM medications and chemotherapy regimens. Additionally, laboratory findings [FBG, glycosylated hemoglobin A1c (HbA1c), leukocyte count, platelet count, hemoglobin, neutrophil count, creatinine, alanine aminotransferase and aspartate aminotransferase] and clinical symptoms during first‐line therapy (including constipation, diarrhea, fatigue, nausea and vomiting) were also extracted to analyze the adverse events of chemotherapy.

The study was carried out in accordance with the Declaration of Helsinki and conformed to the ethical guidelines issued by the Ethics Committee of Xinqiao Hospital, Third Military Medical University (Chongqing, China). The recorded data were anonymized for analysis.

### 2.2 Measurements

T2DM was diagnosed mainly due to an elevated FBG level (>7.0 mmol/L) or random blood glucose (>11.1 mmol/L), a history of diabetes or pharmaceutical treatment for diabetes.^23^ The blood glucose control conditions were evaluated individually by three independent physicians according to the *Standards of care for type 2 diabetes in China.* 23 To eliminate the influence of chemotherapy on blood glucose, the FBG before each chemotherapy cycle was monitored for evaluation.^24^ According to the assessment results of glycemic control during chemotherapy, we categorized patients with T2DM into good control and poor control groups.^23^

An accepted surrogate end point, PFS, was used to estimate the efficacy of chemotherapy in NSCLC patients on the basis of RECIST.^22^ PFS was originally defined as the time from randomization in a clinical trial to objective tumor progression or death. Here, the initial time was modified as the most recent examination of chest CT scan prior to the first treatment (interval between CT scan and chemotherapy not exceeding 2 weeks); the endpoint was determined as tumor progression, death or the date of last follow‐up.

The toxicity assessments involved constitutional symptoms, gastrointestinal symptoms, and laboratory results (hematology and blood chemistry). Side effects related to chemotherapy were graded according to the Common Terminology Criteria for Adverse Events version 4.0.^25^

### 2.3 Statistical analysis

Data are presented as the mean ± standard deviation (SD), median (95% confidence interval, 95% CI), or number (percentage) depending on the types of variables. The differences in data for baseline characteristics and data related to adverse reactions between the two groups (poor glycemic control and good glycemic control) were analyzed using a chi‐square test or Student's *t* test. PFS was estimated using the Kaplan‐Meier method. Multivariate survival analysis was performed using Cox regression analysis. This analysis was used to evaluate the impact of blood glucose control on PFS in the presence of other potentially confounding variables. Statistical differences were regarded as significant with a p value less than 0.05.

All of the data analyses were performed using GraphPad Prism version 7 (GraphPad Software, San Diego, CA) and SPSS 20.0 statistical software (SPSS, Inc).

## 3 RESULTS

### 3.1 Baseline characteristics

In total, 200 advanced NSCLC patients without T2DM and 191 participants with T2DM were included in the final analysis of this study. The baseline characteristics of the investigated patients are listed in Table 1. Overall, most patients with T2DM were male (86.4%) and had an ECOG PS of 0 or 1 (87.4%), with a mean age of 61.5 ± 8.1 years and a mean BMI of 23.8 ± 3.0 kg/m^2^. Almost two thirds of patients (62.3%) had metastatic lesions. More than half of the patients developed other complications at the time of cancer diagnosis, including hypertension, coronary heart disease and chronic obstructive pulmonary diseases.

**Table 1:** Baseline characteristics of patients with advanced NSCLC

Characteristics	None (200)	Patients with T2DM (191)				*P* *
		Poor (84)	Good (107)	All (191)	*P* **	
Age, mean years ± SD	59.2 ± 10.1	60.5 ± 8.0	62.4 ± 8.2	61.5 ± 8.1	0.119	.013
Sex					.059	.041
Male, n (%)	157 (78.5%)	77 (91.7%)	88 (82.2%)	165 (86.4%)		
Female, n (%)	43 (21.5%)	7 (8.3%)	19 (17.8%)	26 (13.6%)		
BMI (kg/m^2^)	22.9 ± 2.5	23.7 ± 2.7	23.9 ± 3.3	23.8 ± 3.0	.931	.001
TNM Stages					.964	.236
III, n (%)	66 (33.0%)	28 (33.3%)	36 (33.6%)	74 (38.7%)		
IV, n (%)	134 (77.0%)	56 (66.7%)	71 (66.4%)	117 (61.3%)		
Pathological type					.704	.189
Adenocarcinoma, n (%)	121 (60.5%)	44 (52.4%)	59 (55.1%)	103 (53.9%)		
Squamous cell carcinoma, n (%)	79 (39.5%)	40 (47.6%)	48 (44.9%)	88 (46.1%)		
ECOG PS					.130	.063
0‐1, n (%)	186 (93.0%)	70 (83.3%)	97 (90.7%)	167 (87.4%)		
2, n (%)	14 (7.0%)	14 (16.7%)	10 (9.3%)	24 (12.6%)		
Complications					.473	<.001
No, n (%)	154 (77.0%)	38 (45.2%)	54 (50.5%)	92 (48.2%)		
Yes, n (%)	46 (23.0%)	46 (54.8%)	53 (49.5%)	99 (51.8%)		
Smoking history					.640	.002
Never, n (%)	97 (48.5%)	23 (27.4%)	36 (33.6%)	59 (30.9%)		
Ever, n (%)	81 (40.5%)	48 (57.1%)	55 (51.4%)	103 (53.9%)		
Current, n (%)	22 (11.0%)	13 (15.5%)	16 (15.0%)	29 (15.2%)		
Drinking status					.048	.010
Never, n (%)	153 (76.5%)	61 (72.6%)	72 (67.3%)	133 (69.6%)		
Ever, n (%)	40 (20.0%)	10 (11.9%)	26 (24.3%)	36 (18.9%)		
Current, n (%)	7 (3.5%)	13 (15.5%)	9 (8.4%)	22 (11.5%)		
Chemotherapy(Platinum +)					.205	<.001
Docetaxel, n (%)	15 (7.5%)	16 (19.0%)	18 (16.8%)	34 (17.8%)		
Pemetrexed, n (%)	35 (17.5%)	8 (8.5%)	22 (20.6%)	30 (15.7%)		
Paclitaxel, n (%)	122 (61.0%)	29 (34.5%)	25 (23.4%)	54 (28.3%)		
Gemcitabine, n (%)	20 (10.0%)	17 (20.2%)	23 (21.5%)	40 (21.0%)		
Others, n (%)	8 (4.0%)	14 (16.7%)	19 (17.7%)	33 (17.2%)		

Abbreviations: BMI, Body mass index; ECOG PS, Eastern Cooperative Oncology Group performance status; NSCLC, non‐small cell lung cancer; SD, standard deviation; T2DM, type 2 diabetes mellitus.

* *P* value indicated the difference between patients with type 2 diabetes mellitus and those without.

** *P* value indicated the difference between the good blood glucose control group (good) and the poor blood glucose control group (poor).

Among all subjects with T2DM, 107 subjects were classified into the good control group (good glycemic control), and the others (n = 84) were classified into the poor control group (poor glycemic control) based on the monitoring results of FBG during chemotherapy (6.1 ± 1.1 mmol/L vs 10.2 ± 2.8 mmol/L, *P* < .0001). Moreover, the maximum HbA1c values available from a total of 97 subjects during chemotherapy were collected as well. The mean of the maximum HbA1c values was also lower than that of the poor control group (7.7% ± 1.38% vs 8.6% ± 2.52%, *P* < .001).

There were no statistically significant differences in patients between the poor control group and good control group in terms of age (*P* = .119), sex (*P* = .059), BMI (*P* = .931), TNM stages (*P* = .964), complications (*P* = .473), ECOG PS (*P* = .130), pathological type (*P* = .704), smoking history (*P* = .640) and first‐line chemotherapy schemes (*P* = .205). However, drinking status was slightly different (*P* = .048).

### 3.2 Survival

The potential known factors affecting prognosis, such as sex, age, smoking history, drinking status, BMI, tumor stage, complications except T2DM, ECOG PS and pathological type, were included in the univariate and multivariate Cox regression analyses. A multivariate Cox regression analysis including all patients in the study revealed that preexisting T2DM was an independent prognostic factor for PFS (hazard ratio: 1.561, 95% CI: 1.223‐1.993, *P* ˂ .001), even though there existed significant differences between patients with and without T2DM in terms of age (*P* = .013), sex (*P* = .041), BMI (*P* = .001), other complications except T2DM (*P* ˂ .001), smoking history (*P* = .002), drinking status (*P* = .010) and chemotherapy regimens (*P* ˂ .001) at baseline. As listed in Table 2, the results also revealed that glycemic status had a substantial impact on the PFS of NSCLC patients with T2DM undergoing platinum‐based chemotherapy (hazard ratio: 1.738, 95% CI: 1.364‐2.663, *P* = .0003). After adjustment for multiple potentially confounding factors, poor glycemic control remained the most significant reason for poor survival (hazard ratio: 1.769, 95% CI: 1.259‐2.486, *P* = .001).

**Table 2:** Univariate and multivariate analyses of potential prognostic variables for survival in NSCLC patients with T2DM

Variables	Reference	Univariate analyses		Multivariate analyses	
		*P* value	HR (95% CI)	*P* value	HR (95% CI)
Age (≥60)	Age (<60)	.714	1.061(0.773‐1.455)	.827	0.963(0.685‐1.353)
Male	Female	.437	1.202(0.775‐1.864)	.285	1.370(0.769‐2.440)
BMI (≥25)	BMI (<25)	.484	1.127(0.798‐1.591)	.720	1.065(0.753‐1.507)
TNM: IV	TNM: III	.248	1.214(0.877‐1.675)	.369	0.849(0.594‐1.213)
Squamous cell carcinoma	Adenocarcinoma	.983	0.997(0.729‐1.363)	.485	0.883(0.623‐1.252)
ECOG PS: 2	ECOG PS: 0‐1	.250	1.311(0.719‐2.207)	.331	1.273(0.782‐2.070)
Tobacco use	No use	.718	0.941(0.673‐1.316)	.485	0.861(0.565‐1.311)
Alcohol use	No use	.152	0.781(0.561‐1.086)	.330	0.829(0.568‐1.209)
Complications	None	.172	1.241(0.908‐1.695)	.212	1.228(0.886‐1.730)
Poor control of blood glucose	Good control of blood glucose	.0003	1.738(1.364‐2.663)	.001	1.769(1.259‐2.486)

Abbreviations: BMI, body mass index; CI, confidence interval; ECOG PS, Eastern Cooperative Oncology Group performance status; HR, hazard ratio; NSCLC, non‐small cell lung cancer; T2DM, type 2 diabetes mellitus.

**Figure 1:**
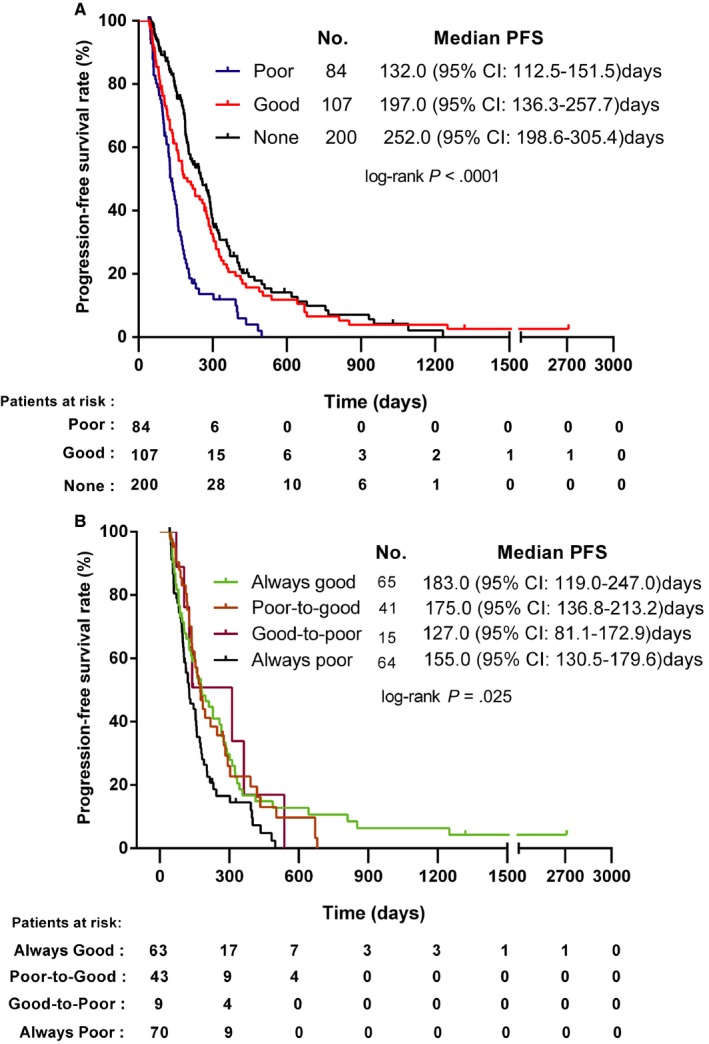
Kaplan‐Meier plots of progression‐free survival in patients with non‐small cell lung cancer according to the glycemic control during chemotherapy (None: patients without T2DM; Good: patients with good glycemic control; Poor: patients with poor glycemic control; Figure 1A); and according to the changes of glycemic control before and during chemotherapy (always good: good glycemic control before and during chemotherapy; poor‐to‐good: poor glycemic control before chemotherapy, good glycemic control during chemotherapy; good‐to‐poor: good glycemic control before chemotherapy, poor glycemic control during chemotherapy; always poor: poor glycemic control before and during chemotherapy; Figure 1B) (HR = hazard ratio; CI = confidence intervals)

The median PFS of the 200 patients without T2DM was much longer than that of the patients with T2DM [252.0 (95% CI: 198.6‐305.4) days and 157.0 (95% CI: 134.0‐180.0) days; *P* ˂ .0001]. The subgroup analysis revealed that the patients without T2DM exhibited better survival than the T2DM patients, regardless of their glycemic control status [ie, good or bad, 252.0 (95% CI: 198.6‐305.4) days vs 197.0 (95% CI: 136.3‐257.7) days, *P* = .1234; 252.0 (95% CI: 198.6‐305.4) days vs 132.0 (95% CI: 112.5‐151.5) days, *P* ˂ .0001; respectively]. Furthermore, analysis of the whole cohort revealed that the median PFS of patients with good glycemic control was significantly longer than that of patients with poor glycemic control [197.0 (95% CI: 136.3‐257.7) days vs 132.0 (95% CI: 112.5‐151.5) days, *P* = .0003)]. The Kaplan‐Meier survival analysis clearly exhibited a significant trend toward a longer PFS among patients without T2DM and with good metabolic control in comparison with those with poor metabolic control (Figure 1A).

To confirm our results, the FBG before the first chemotherapy cycle was also collected in totally 185 of 191 patients. Based on the changes in FBG level before and during chemotherapy, these 185 patients were classified into four groups: always good glycemic control (n = 63), poor‐to‐good glycemic control (n = 43), good‐to‐poor glycemic control (n = 9) and always poor glycemic control (n = 70). The longest PFS was found in patients with always good glycemic control [183.0 (95% CI: 119.0‐247.0) days], followed by poor‐to‐good [175.0 (95% CI: 136.8‐213.2) days], always poor [155.0 (95% CI: 130.5‐179.6) days] and good‐to‐poor glycemic control [127.0 (95% CI: 81.1‐172.9) days], as shown in Figure 1B.

**Figure 2:**
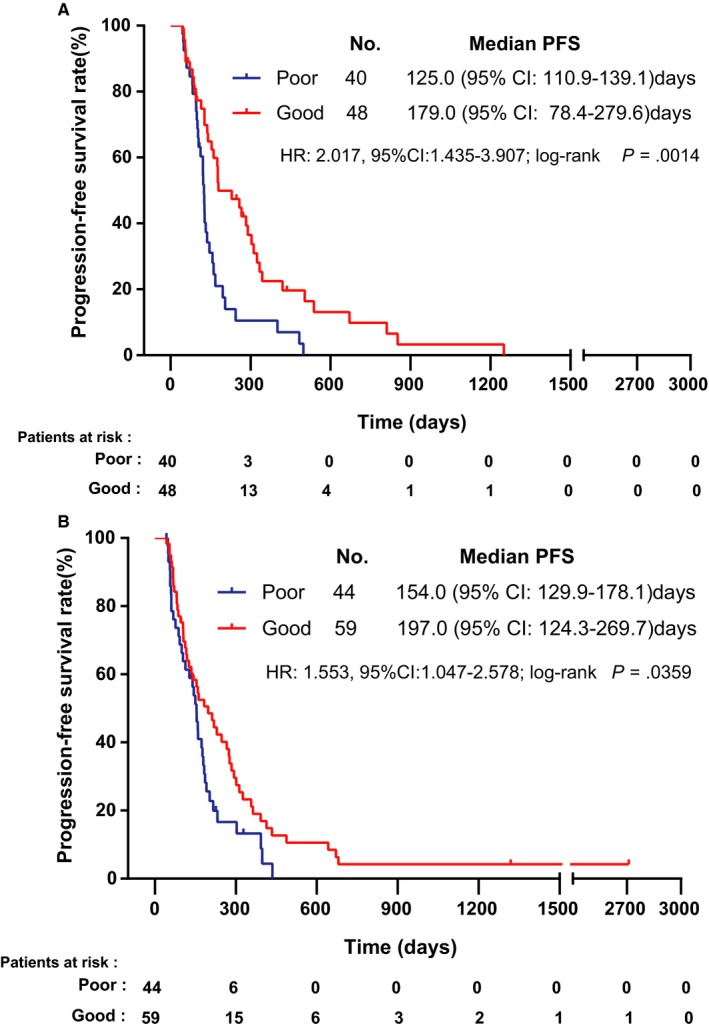
Kaplan‐Meier plots of progression‐free survival in type 2 diabetes mellitus patients with adenocarcinoma (Figure 2A) or squamous cell carcinoma (Figure 2B) according to the glycemic control state during chemotherapy (HR = hazard ratio; CI = confidence interval)

When separately analyzing patients with different pathological subtypes of cancer, the median PFS values of squamous cell lung carcinoma and adenocarcinoma patients were 146.0 (95% CI: 116.1‐175.9) days and 159.0 (95% CI: 132.5‐185.5) days, respectively. Similarly, we also found that among patients with squamous cell carcinoma, those with poor glycemic control had a significantly worse median PFS than those with good glycemic control [125.0 (95% CI: 110.9‐139.1) days vs 179.0 (95% CI: 78.4‐279.6) days, *P* = .0014; Figure 2A)]; the same pattern was observed among those with adenocarcinoma [154.0 (95% CI: 129.9‐178.1) days vs 197.0 (95% CI: 124.3‐269.7) days, *P* = .0359; Figure 2B)].

**Figure 3:**
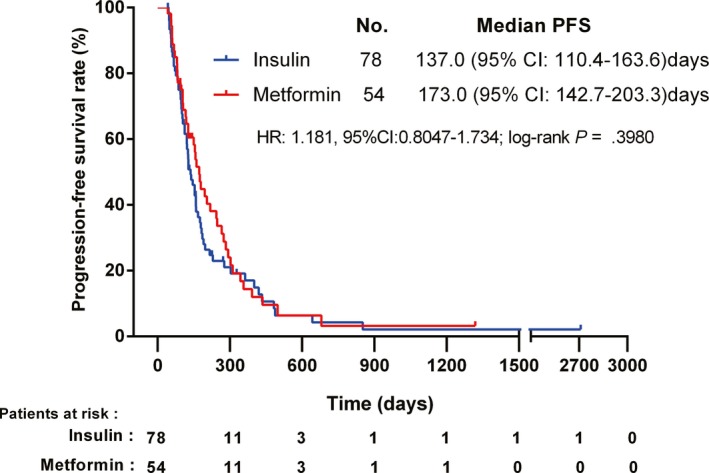
Kaplan‐Meier plots of progression‐free survival in type 2 diabetes mellitus patients with non‐small cell lung cancer according to different glucose‐lowering therapies (insulin and metformin) during chemotherapy (HR = hazard ratio; CI = confidence interval)

We constructed another model to compare the influence of antidiabetic treatment (metformin and insulin) on prognosis, for the two drugs were the most representative and have drawn much argument on survival.^26^, ^27^ There were 54 patients using metformin‐containing antidiabetic regimens without insulin (metformin group) and 78 patients using insulin‐contained antidiabetic regimens without metformin (insulin group). The median PFS was 173.0 (95% CI: 142.7‐203.3) days and 137.0 (95% CI: 110.4‐163.6) days in the metformin group and insulin group, respectively. As shown in Figure 3, no statistically significant differences were observed in PFS among T2DM patients undergoing either of the two antidiabetic regimens (*P* = .3980). A further subgroup analysis revealed no difference in PFS between patients with metformin and insulin, regardless of their glycemic state [good control with metformin vs good control with insulin: 159.0 (95% CI: 129.7‐188.3)days vs 128.0 (95% CI: 108.6‐147.4)days, *P* = .1671; poor control with metformin vs poor control with insulin: 127.0 (95% CI: 76.8‐177.2)days vs 154.0 (95% CI: 97.3‐210.7)days; *P* = .6156, respectively].

### 3.3 Adverse events

All 191 patients were available for safety analysis. The treatment‐related adverse events are shown in Table 3. Although the treatment time was relatively longer, the overall incidence and severity of all grades of side effects in patients with good glycemic control was comparable to that of poor glycemic control in general (all *P* ˃ .05). The major side effect in both groups was decreased hemoglobin, occurring in approximately 90% of all subjects. Grade 3 or more toxicities, including severe nausea, vomiting, diarrhea and constipation, were observed in only a few patients with poor glycemic control. None of the patients experienced grade 3 or worse toxicity in the good glycemic group. No treatment‐related death occurred.

**Table 3:** Common adverse events related to chemotherapy

Events (n, %)	Poor control (n = 84)		Good control (n = 107)		*P* value*
	All grades	≥Grade3	All grades	≥Grade3	
Laboratory results					
Leukocytes	32(38.1%)	6(7.1%)	43(40.2%)	11(10.3%)	.769
Neutrophils	29(34.5%)	9(10.7%)	46(43.0%)	15(14.0%)	.234
Hemoglobin	74(88.1%)	14(16.7%)	97 (90.6%)	12(11.2%)	.309
Platelets	46(54.8%)	4(4.8%)	60(56.1%)	7(6.5%)	.856
ALT	25(29.8%)	0(%)	39(36.4%)	0(%)	.131
AST	29(34.5%)	1(1.2%)	14(13.1%)	0(%)	.064
Creatinine	4(4.8%)	0(%)	7(6.5%)	0(%)	.600
Clinical symptoms					
Fatigue	24(28.6%)	0	31(29%)	0	.952
Anorexia	21(25.0%)	0	27(25.2%)	0	.971
Nausea	35(41.7%)	1(1.2%)	53(49.5%)	0	.279
Vomiting	12(14.3%)	1(1.2%)	14(13.1%)	0	.810
Diarrhea	4(4.8%)	3(3.6%)	3(2.8%)	0	.475
Constipation	17(20.2%)	1(1.2%)	23(21.5%)	0	.832

Abbreviations: ALT, aspartate aminotransferase; AST, alanine aminotransferase.

* *P* value indicated the significance of the difference among all grades of adverse events in patients between the good blood glucose control group (good control) and the poor blood glucose control group (poor control).

## 4 DISCUSSION

DM is a major concern in China and worldwide and is a risk factor for various tumors as well.^28^, ^29^ To the best of our knowledge, previous studies focused on the prognostic significance of preexisting diabetes in patients with NSCLC, but have not reached a consensus.^30^, ^31^ One reason might be that preexisting diabetes or baseline FBG levels are not the most decisive factor influencing prognosis, but the blood glucose control level during chemotherapy might be a factor. Therefore, we initiated this study to investigate the relationship between blood glucose control and the outcome of patients with advanced NSCLC receiving first‐line platinum‐based doublets chemotherapy.

Here, we observed that T2DM patients with a better glycemic control had a longer PFS, in either squamous cell carcinoma or adenocarcinoma or as a whole. Also, good glycemic control was independently related to favorable prognosis in the multivariate model. Moreover, the Kaplan‐Meier method showed that the blood glucose control during chemotherapy seemed to affect the survival more than the glycemic control or baseline FBG before chemotherapy did. These findings underlined the potential benefit of achieving good metabolic control during chemotherapy to improve the outcome of T2DM patients with NSCLC receiving platinum‐based chemotherapy. Notably, the number of NSCLC subjects with T2DM enrolled in this study was higher than the number of patients included in previous studies.^9^, ^10^, ^30^

As for the biological mechanism of unfavorable survival in T2DM patients with poor blood glucose control, several possible explanations have been proposed as follows. First, insulin resistance (a basic feature of T2DM) may promote cancer cell proliferation, differentiation and metastasis by activating the insulin‐like growth factor‐1 (IGF‐1) receptor signaling pathway.^32^, ^33^ On the other hand, cancer cells are characterized by an accelerated metabolism, high glucose requirements and an increased glucose uptake.^34^, ^35^ A hyperglycemic environment may facilitate the proliferation of cancer cells for this reason. Numerous studies in patients and mouse models have also indicated that high expression levels of glycolytic enzymes contribute to worse survival.^31^, ^36^, ^37^ Additionally, high glucose levels can induce the upregulation of epidermal growth factor expression and the transactivation of the epidermal growth factor receptor, subsequently activating downstream signaling pathways, such as ERK and STAT 3, thus promoting cancer cell proliferation.^38^, ^39^, ^40^ Lastly, the hyperglycemic environment markedly enhances cellular reactive oxygen species and confers P‐glycoprotein‐mediated multidrug resistance to chemotherapeutics.^41^

The administration of antidiabetic agents, including insulin (insulin analogs) and oral hypoglycemic drugs, has been reported to affect the development and progression of carcinomas.^42^, ^43^ Traditionally, metformin plays as a potential antitumor role through molecular mechanisms of the mTOR signaling pathway and the ATM/LKB1/AMPK axis,^44^ whereas insulin or insulin analogs stimulate mitogenesis and the proliferation of tumor cells by activating the IGF‐1 receptor signaling pathway.^45^ However, the effect of antidiabetic drugs on NSCLC patients undergoing platinum‐based chemotherapy has caused a heated discussion in the clinic.^46^, ^47^ In line with other reports,^10^, ^47^ our results showed no significant difference in PFS among groups with the two different hypoglycemic treatments mentioned above, and these findings was obtained for the whole and in the subgroup (good or poor glycemic state) analysis, which might indicate that the changes of PFS did not depend on the strategies used to lower the glucose level, but did depend on the state of glycemic control.

This study has some inevitable disadvantages. First, it was retrospective and some data were missing (especially HbA1c). In addition, our findings need to be confirmed at other centers and in other race/ethnicity groups. Furthermore, certain histological types of NSCLC (large cell lung carcinoma) in patients with T2DM were not included in our study. Finally, we did not collect the overall survival of patients, because different second‐line treatments may have affected the survival markedly.^48^

In conclusion, good glycemic control seemed to be a favorable prognostic factor in T2DM patients with NSCLC at the advanced stage receiving first‐line platinum‐based doublet chemotherapy based on the observed longer PFS in our study. Since increased blood glucose control could enhance the efficacy of chemotherapy without additional adverse events, much attention should be paid to it in these patients clinically. However, considering the limitations of this study, more studies (especially prospective studies) are necessary to validate and update our conclusions.

## CONFLICT OF INTEREST

The authors declare no conflict of interest.

## Data Availability

The data that support the findings of this study are available on request from the corresponding author. The data are not publicly available due to privacy or ethical restrictions.
